# Pirfenidone Sensitizes NCI-H460 Non-Small Cell Lung Cancer Cells to Paclitaxel and to a Combination of Paclitaxel with Carboplatin

**DOI:** 10.3390/ijms23073631

**Published:** 2022-03-26

**Authors:** Helena Branco, Júlio Oliveira, Catarina Antunes, Lúcio L. Santos, Maria Helena Vasconcelos, Cristina P. R. Xavier

**Affiliations:** 1 i3S—Instituto de Investigação e Inovação em Saúde, University of Porto, Rua Alfredo Allen 208, 4200-135 Porto, Portugal; hbranco@ipatimup.pt (H.B.); catarina.teixeira.antunes@gmail.com (C.A.); 2 Cancer Drug Resistance Group, IPATIMUP—Institute of Molecular Pathology and Immunology, University of Porto, Rua Alfredo Allen 208, 4200-135 Porto, Portugal; 3 Experimental Pathology and Therapeutics Group, IPO—Instituto Português de Oncologia, Rua Dr. António Bernardino de Almeida 865, 4200-072 Porto, Portugal; julio.oliveira@ipoporto.min-saude.pt (J.O.); lucios@ufp.pt (L.L.S.); 4 ICBAS-UP—School of Medicine and Biomedical Sciences, University of Porto, Rua de Jorge Viterbo Ferreira 228, 4050-313 Porto, Portugal; 5 Department of Biological Sciences, FFUP—Faculty of Pharmacy, University of Porto, Rua de Jorge Viterbo Ferreira 228, 4050-313 Porto, Portugal

**Keywords:** lung cancer, drug repurposing, chemotherapy, paclitaxel, pirfenidone

## Abstract

Pirfenidone, an antifibrotic drug, has antitumor potential against different types of cancers. Our work explored whether pirfenidone sensitizes non-small cell lung cancer (NSCLC) cell lines to chemotherapeutic treatments. The cytotoxic effect of paclitaxel in combination with pirfenidone against three NSCLC cell lines (A549, NCI-H322 and NCI-H460) was evaluated using the sulforhodamine B assay. The effects of this combination on cell viability (trypan blue exclusion assay), proliferation (BrdU incorporation assay), cell cycle (flow cytometry following PI staining) and cell death (Annexin V-FITC detection assay and Western blot) were analyzed on the most sensitive cell line (NCI-H460). The cytotoxic effect of this drug combination was also evaluated against two non-tumorigenic cell lines (MCF-10A and MCF-12A). Finally, the ability of pirfenidone to sensitize NCI-H460 cells to a combination of paclitaxel plus carboplatin was assessed. The results demonstrated that pirfenidone sensitized NCI-H460 cells to paclitaxel treatment, reducing cell growth, viability and proliferation, inducing alterations in the cell cycle profile and causing an increase in the % of cell death. Remarkably, this combination did not increase cytotoxicity in non-tumorigenic cells. Importantly, pirfenidone also sensitized NCI-H460 cells to paclitaxel plus carboplatin. This work highlights the possibility of repurposing pirfenidone in combination with chemotherapy for the treatment of NSCLC.

## 1. Introduction

Despite recent breakthroughs in lung cancer diagnosis and management, this disease remains the second most commonly diagnosed cancer and the leading cause of cancer-associated death worldwide [1]. Lung cancer is a heterogenous group of diseases that can be classified into two major histologic types: small cell lung cancer (SCLC) and non-small cell lung cancer (NSCLC). Particularly, NSCLC accounts for roughly 85% of the cases and, depending on the tissue of origin, is categorized into three main histologic subtypes: adenocarcinoma (40%); squamous cell carcinoma (25–30%); and large cell carcinoma (5–10%) [2].

Regardless of the histological type, lung cancer treatment is primarily based on five strategies: surgery, radiotherapy, chemotherapy, molecularly targeted therapy and immunotherapy [3]. These strategies can be applied independently or in combinatorial regimens, contributing to an increasingly customized clinical management. Surgery is considered the cornerstone of early-stage NSCLC (I, II or IIIA) treatment. Perioperative platinum-based chemotherapy (e.g., Paclitaxel plus Carboplatin/Cisplatin) has been applied to improve overall survival rate, lowering post-operative risk of relapse [4,5,6]. Nevertheless, over 60% of patients are diagnosed at a locally advanced or metastatic stage of the disease, at which point surgical resection is no longer feasible [1,7]. The introduction of immune or targeted therapies into lung cancer management has resulted in a significantly improved prognosis for patients expressing higher levels of PD-L1 or carrying molecularly targetable alterations, respectively [8,9,10]. However, despite the encouraging results associated with these two therapeutic modalities, a large proportion of patients do not meet the criteria for single-agent immune or target therapy, or acquire mechanisms of drug resistance; in these cases, therapeutic regimens with platinum-based chemotherapy are the available treatment options [11,12,13]. Hence, chemotherapy remains at the center of lung cancer management, and the development of new and more effective therapeutic combinations is of great interest.

Drug repurposing strategies identify new clinical applications for drugs that are already approved or under investigation for the treatment of other medical conditions [14,15]. This innovative process has several advantages over traditional drug discovery, as it eliminates the majority of the steps associated with early pharmacological development, such as safety, toxicity, pharmacokinetic and pharmacodynamic studies [16,17,18]. Indeed, drug repurposing significantly reduces the time and costs associated with traditional drug discovery, allowing for a faster transition from bench to bedside [19,20].

Pirfenidone, which is an anti-fibrotic, anti-inflammatory and antioxidant drug approved by the European Medicines Agency (EMA) and the United States Food and Drug Administration (FDA) for the treatment of idiopathic pulmonary fibrosis [21,22], has recently demonstrated antitumor potential against several types of cancer. Indeed, pirfenidone suppresses cancer cell growth, viability and proliferation, as well as induces tumor cell death, alters the cell cycle profile and/or interferes with multiple cancer-associated signaling pathways in numerous types of cancers, such as prostate and pancreatic cancer, hepatocellular carcinoma, mesothelioma and glioma [23,24,25,26,27]. Furthermore, as an antifibrotic drug, pirfenidone has also been shown to modulate the tumor microenvironment by interfering with cancer-associated fibroblasts in pancreatic and breast cancer models [28,29]. Regarding NSCLC, Marwitz S. et al. (2020) recently demonstrated that pirfenidone reduces tumor cell viability, proliferation and migration by inducing a G0/G1 cell cycle arrest and downregulating the TGF-β/SMAD signaling pathway [30]. Furthermore, some studies found that pirfenidone interferes with NSCLC tumor cell motility through the epithelial-to-mesenchymal transition process [31,32]. 

Interestingly, pirfenidone in combination with other therapeutic modalities has also been explored. For instance, Kozono S. et al. (2013) used an in vivo pancreatic cancer model to demonstrate that pirfenidone increases gemcitabine’s ability to suppress tumor growth and dissemination [28]. Likewise, Choi S. et al. (2015) showed that pirfenidone sensitizes radio-resistant Lewis lung carcinoma tumors to the combined treatment of radiotherapy with sunitinib [33]. Polydorou C. et al. (2017) verified that pirfenidone increases the sensitivity of two orthotopic breast cancer models to doxorubicin treatment [34]. In these last two studies, pirfenidone’s effect was correlated with its ability to modulate the tumor extracellular-matrix through the inhibition of TGF-β-induced collagen deposition. In NSCLC, the combined treatment of pirfenidone with cisplatin had a synergistic effect against both tumor cells and cancer-associated fibroblasts [35]. Recently, Qin W. et al. (2020) explored the anti-tumor effect of pirfenidone in combination with an anti-PD-L1 antibody in an in vivo NSCLC model, demonstrating pirfenidone’s ability to increase both T cell infiltration and the expression of cytokines and chemokines [36]. Nevertheless, the sensitizing effect of pirfenidone to other chemotherapeutic drugs currently being used in the clinic for NSCLC treatment has not been yet explored. 

Our work intended to understand whether pirfenidone could sensitize NSCLC cells to treatment with paclitaxel, which is a chemotherapeutic drug currently used in the treatment of NSCLC patients. For that, the cell growth inhibitory effect of the combined treatment of paclitaxel with pirfenidone was evaluated in three human NSCLC cell lines—the adenocarcinoma A549 and NCI-H322 cell lines, and the large cell carcinoma NCI-H460 cell line. In addition, the effects of this combined drug treatment on cell viability, cell proliferation, cell cycle profile, cell death and on the expression levels of apoptotic-related proteins were further analyzed for the most sensitive cell line (NCI-H460). Importantly, the cytotoxic effect of this combined drug treatment on two non-tumorigenic cell lines, MCF-10A and MCF-12A, was also determined. Finally, the sensitizing effect of pirfenidone to the drug combination consisting of paclitaxel plus carboplatin, which is currently used in clinical practice, was also assessed in NCI-H460 cells.

## 2. Results

### 2.1. The Combined Treatment of Paclitaxel with Pirfenidone Reduces the Growth of Three Human NSCLC Cell Lines

To investigate the tumor cell growth inhibitory activity of the combined treatment of paclitaxel with pirfenidone, the cytotoxic effect of each individual drug—paclitaxel or pirfenidone—was first evaluated, using the sulforhodamine B (SRB) assay. For that, three human NSCLC cell lines, A549, NCI-H322 and NCI-H460, were treated with five serial dilutions of each drug individually for 48 h, and the GI_50_ concentrations (that cause 50% of cell growth inhibition) were assessed by an interpolation on the acquired dose–response curves (Supplementary Figure S1). The results, presented in Table 1, demonstrated that, as expected, all three NSCLC cell lines presented higher sensitivity to paclitaxel treatment than to pirfenidone. Indeed, as predicted, the GI_50_ concentrations obtained for pirfenidone in the NSCLC cell lines were significantly higher than those obtained for the chemotherapeutic drug paclitaxel, as pirfenidone is not a drug commercially approved for cancer treatment.

**Figure 1:**
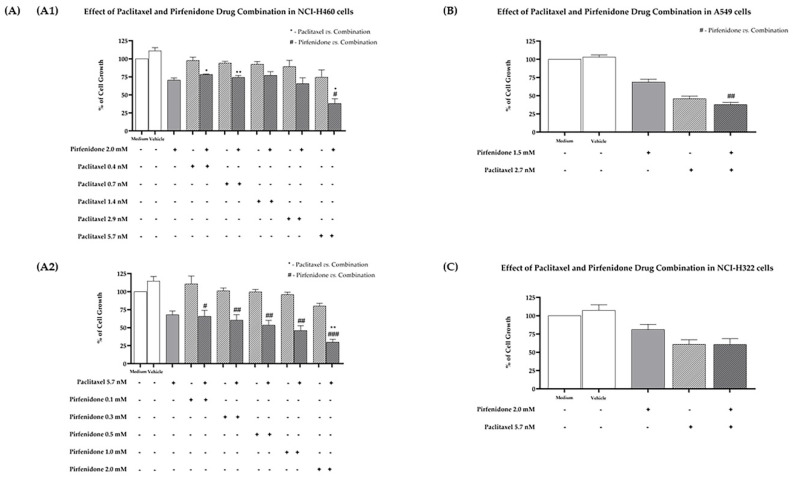
Effect of the combined treatment of paclitaxel with pirfenidone on the % of cell growth of NCI-H460, A549 and NCI-H322 non-small cell lung cancer (NSCLC) cell lines, assessed by the SRB assay. (**A**) NCI-H460 cells treated for 48 h with drug combinations consisting of: 2.0 mM pirfenidone with five serial dilutions of paclitaxel (**A1**); or 5.7 nM paclitaxel with five serial dilutions of pirfenidone (**A2**). (**B**) A549 cells treated for 48 h with a drug combination consisting of 1.5 mM pirfenidone and 2.7 nM paclitaxel. (**C**) NCI-H322 cells treated for 48 h with a drug combination consisting of 2.0 mM pirfenidone and 5.7 nM paclitaxel. The effect of the vehicle at the highest concentration tested was also analyzed. Results are presented as % of cell growth and are the mean ± SEM of at least three independent experiments. * or # *p* < 0.05, ** or ## *p* < 0.01 and ### *p* < 0.001.

Furthermore, the effect of the combined treatment of paclitaxel with pirfenidone on the % of cell growth on the three human NSCLC cell lines under study was evaluated using the SRB assay. The NCI-H460 cell line was treated for 48 h with drug combinations consisting of: (1) pirfenidone at 2 mM with increasing concentrations of paclitaxel (Figure 1(A1)); or (2) paclitaxel at 5.7 nM with increasing concentrations of pirfenidone (Figure 1(A2)). Results revealed that the combined treatment of paclitaxel 5.7 nM with pirfenidone 2.0 mM statistically significantly reduced the % of NCI-H460 cell growth, when compared with treatment with each drug individually. These results indicate that pirfenidone sensitizes NCI-H460 cells to paclitaxel treatment. 

Next, these results were validated in two other NSCLC cell lines. The A549 cells were treated for 48 h with the drug combination consisting of paclitaxel 2.7 nM and pirfenidone 1.5 mM. The results presented in Figure 1B demonstrate that, even though the combined treatment presented a clear cytotoxic effect against this cancer cell line, this effect was not statistically significant when compared to treatment with each drug individually. The same outcome was observed when NCI-H322 cells were treated for 48 h with paclitaxel 5.7 nM and pirfenidone 2 mM (concentrations that achieved the best results in the NCI-H460 cells). Therefore, we decided to proceed our studies in the NCI-H460 cells.

### 2.2. The Combined Treatment of Paclitaxel with Pirfenidone Efficiently Reduces NCI-H460 Cell Viability and Proliferation

**Figure 2:**
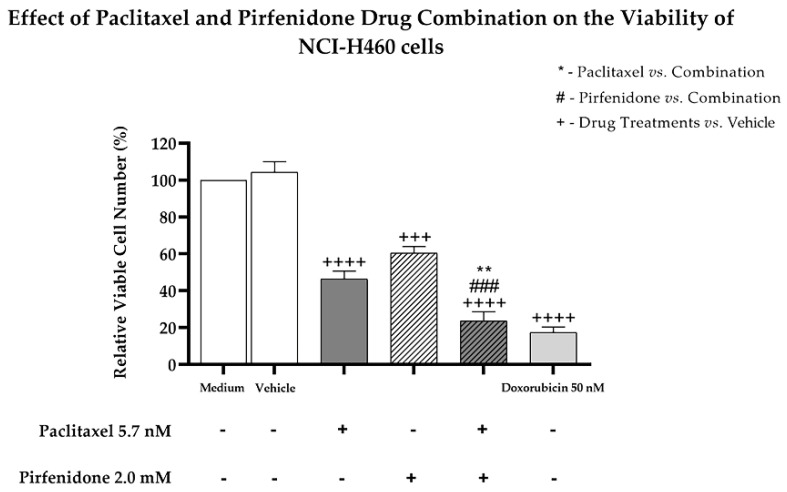
Effect of the combined treatment of paclitaxel with pirfenidone on the relative viable cell number of NCI-H460 cells, assessed by the trypan blue exclusion assay. Cells were treated for 48 h with 5.7 nM paclitaxel and 2 mM pirfenidone, either alone or in combination. The effect of the vehicle at the highest concentration tested in the drug treatments was also analyzed. Doxorubicin (50 nM) was used as a positive control. Results are the mean ± SEM of at least three independent experiments. ** *p* < 0.01, ### or +++ *p* < 0.001 and ++++ *p* < 0.0001.

Considering the previous results, we then evaluated the effect of the combined treatment of paclitaxel with pirfenidone on the viability and proliferation of NCI-H460 cells, using the trypan blue exclusion assay and the bromodeoxyuridine (BrdU) incorporation assay, respectively. For that, NCI-H460 cells were treated for 48 h with the combined treatment consisting of 5.7 nM paclitaxel with 2.0 mM pirfenidone, as well as with each drug individually. The vehicle at the highest concentration tested was used as a negative control. Doxorubicin (50 nM) was used as a positive control. Figure 2 demonstrates that, as expected, doxorubicin statistically significantly reduced NCI-H460 cell viability. The NCI-H460 cells treated with either paclitaxel or pirfenidone statistically significantly reduced the % of viable cell number. Importantly, the combined treatment of paclitaxel with pirfenidone statistically significantly reduced NCI-H460 cell viability more efficiently than the treatment with each drug individually.

**Figure 3:**
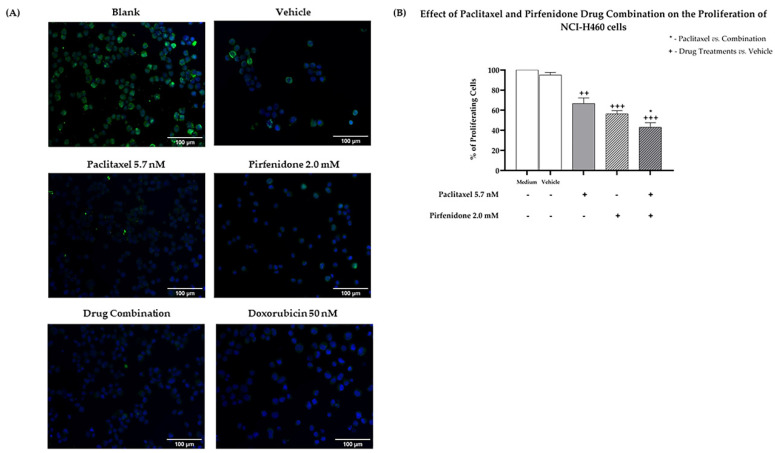
Effect of the combined treatment of paclitaxel with pirfenidone on the proliferation of NCI-H460 cells, assessed by the BrdU incorporation assay. Cells were treated for 48 h with 5.7 nM paclitaxel and 2.0 mM pirfenidone, either alone or in combination. The effect of the vehicle at the highest concentration tested in the drug treatments was also analyzed. Doxorubicin (50 nM) was used as a positive control. (**A**) Representative fluorescence microscopy images of BrdU incorporation (green) and DAPI stained nuclei (blue). Amplification = 200×. (**B**) % of proliferating cells (measured by the % of BrdU incorporating cells). Results are the mean ± SEM of at least three independent experiments. * *p* < 0.05, ++ *p* < 0.01 and +++ *p* < 0.001.

Moreover, results from the BrdU incorporation assay (Figure 3) demonstrated that paclitaxel and pirfenidone alone caused a statistically significant reduction in the % of NCI-H460 proliferating cells. A further decrease in the % of proliferating cells was found following treatment with the combination of paclitaxel with pirfenidone, when compared with paclitaxel alone.

### 2.3. The Combined Treatment of Paclitaxel with Pirfenidone Efficiently Induces Major Alterations on the Cell Cycle Profile of NCI-H460 Cells

**Figure 4:**
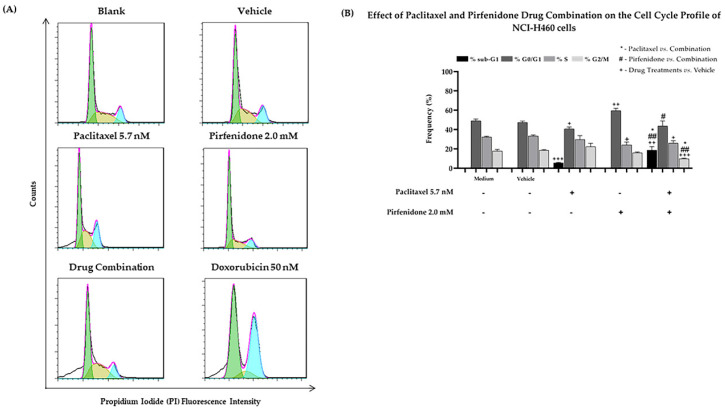
Effect of the combined treatment of paclitaxel with pirfenidone on the cell cycle profile of NCI-H460 cells, assessed by flow cytometry following PI staining. Cells were treated for 48 h with 5.7 nM paclitaxel and 2.0 mM pirfenidone, either alone or in combination. (**A**) Representative cell cycle histograms, and (**B**) frequency of cell cycle phases for each condition. Doxorubicin (50 nM) was used as positive control. The effect of the vehicle at the highest concentration tested in the drug treatments was also analyzed. Results are the mean ± SEM of at least 3 independent experiments. *, # or + *p* < 0.05, ## or ++ *p* < 0.01 and +++ *p* < 0.001.

Taking into consideration the effect of the combined treatment of paclitaxel with pirfenidone on NCI-H460 cell growth, viability and proliferation, we then explored whether these effects could be related to alterations in the cell cycle profile, using flow cytometry following propidium iodide (PI) staining. The NCI-H460 cells were treated for 48 h with the combined treatment consisting of 5.7 nM paclitaxel and 2.0 mM pirfenidone, with each drug alone, with the vehicle at the highest concentration tested (control) and with doxorubicin (50 nM, positive control). Results presented in Figure 4 revealed that, as described in the literature [37,38], doxorubicin caused major alterations in the cell cycle profile. Our results demonstrated that paclitaxel alone caused a statistically significant decrease in the % of cells in the G0/G1 phases of the cell cycle, accompanied by an increase in the sub-G1 cell population. Contrarily, a statistically significant increase in the % of cells in G0/G1, as well as a significant reduction in the % of cells in the S phase of the cell cycle, were found when NCI-H460 cells were treated with 2.0 mM pirfenidone. Regarding the combined treatment of paclitaxel with pirfenidone, our results showed a statistically significant reduction in the % of cells in the G2/M phases of the cell cycle, along with a prominent increase in the % of cells in the sub-G1 phase (suggestive of apoptosis), when compared with the effect of each drug alone. Thus, these results suggest that the combined treatment of paclitaxel with pirfenidone interferes with the cell cycle profile of NCI-H460 cells.

### 2.4. The Combined Treatment of Paclitaxel with Pirfenidone Efficiently Increases NCI-H460 Cell Death

**Figure 5:**
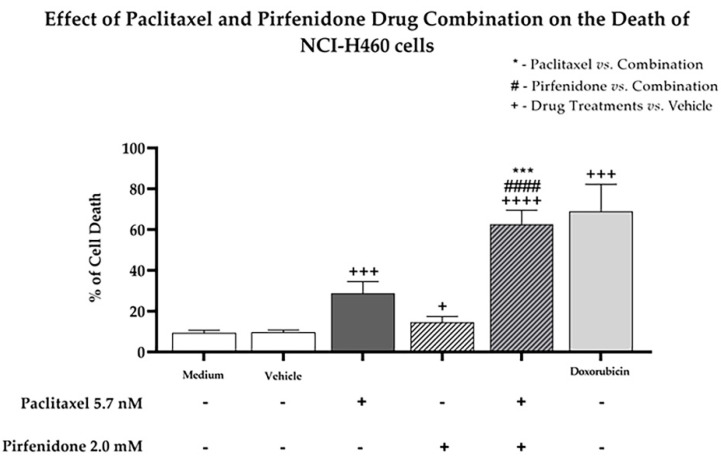
Effect of the combined treatment of paclitaxel with pirfenidone on the levels of NCI-H460 cell death, assessed by flow cytometry following Annexin V-FITC/PI staining. Cells were treated for 48 h with 5.7 nM paclitaxel and 2.0 mM pirfenidone, either alone or in combination. The effect of the vehicle at the highest concentration tested in the drug treatments was also analyzed. Doxorubicin (50 nM) was used as positive control. Results are the mean ± SEM of at least three independent experiments. + *p* < 0.05, *** or +++ *p* < 0.001 and #### or ++++ *p* < 0.0001.

Since our findings pointed to the possibility that the combination of paclitaxel with pirfenidone induced NCI-H460 cell death (due to an increase in the sub-G1 phase of the cell cycle), we then evaluated the effect of this combined treatment on the levels of cell death, using Annexin V-FITC/PI labelling followed by flow cytometry analysis. For that, the NCI-H460 cells were treated for 48 h with the combined treatment consisting of 5.7 nM paclitaxel and 2.0 mM pirfenidone, with each drug individually, with the vehicle at the highest concentration tested (control) and with 50 nM doxorubicin (positive control). The results (Figure 5) demonstrated that both paclitaxel and pirfenidone alone increased NCI-H460 cell death. The combined treatment of 5.7 nM paclitaxel with 2.0 mM pirfenidone was more effective in increasing the % of NCI-H460 cell death, when compared to each individual treatment. These results confirm that the combination of paclitaxel with pirfenidone efficiently induces NCI-H460 cell death.

### 2.5. The Combined Treatment of Paclitaxel with Pirfenidone Causes Alterations in the Expression Levels of Apoptotic-Related Proteins

In order to further confirm the effect of the drug combination on NCI-H460 cell death, we then evaluated the effect of paclitaxel with pirfenidone, either alone or in combination, in the expression levels of some apoptotic-related proteins, such as PARP and caspase-3, by Western blot analysis. For that, NCI-H460 cells were treated for 48 h with the combined treatment (5.7 nM paclitaxel and 2.0 mM pirfenidone), with each drug alone and with the vehicle at the highest concentration tested in the drug treatments (control).

**Figure 6:**
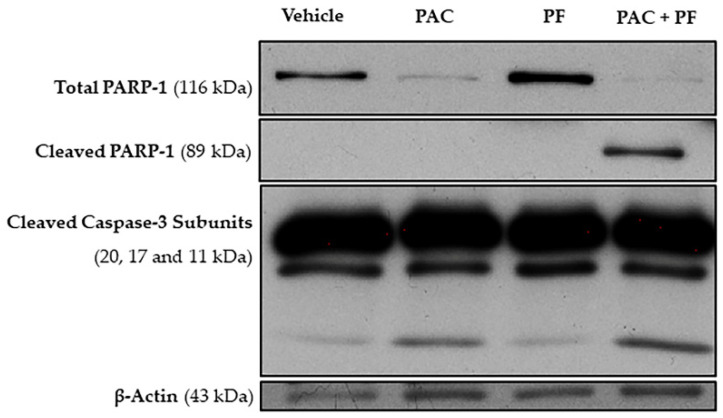
Effect of the combined treatment of paclitaxel with pirfenidone on NCI-H460 cellular expression levels of total PARP-1, cleaved PARP-1 and cleaved caspase-3, assessed by Western blot. Cells were treated for 48 h with 5.7 nM paclitaxel (PAC) and 2.0 mM pirfenidone (PF), either alone or in combination (PAC + PF). The effect of the vehicle at the highest concentration tested in the drug treatments was also analyzed.

The results presented in Figure 6 show a prominent decrease in the expression levels of total poly (ADP-ribose) polymerase 1 (PARP-1), accompanied by an increase in the levels of cleaved PARP-1, when NCI-H460 cells were treated with the drug combination. Moreover, an increase in the expression levels of cleaved caspase-3 was observed following treatment with either paclitaxel alone or with the drug combination. These results corroborate our previous data, which demonstrated a significant increase in the % of NCI-H460 cell death after treatment with the combination of paclitaxel with pirfenidone.

### 2.6. The Combined Treatment of Paclitaxel with Pirfenidone Does Not Cause More Cytotoxic Effect on Human Non-Tumorigenic Cells than Paclitaxel Alone

**Figure 7:**
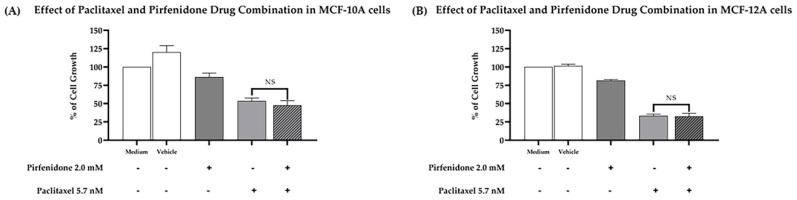
Effect of the combined treatment of paclitaxel with pirfenidone in (**A**) MCF-10A and (**B**) MCF-12A non-tumorigenic cells, assessed by the SRB assay. Cells were treated for 48 h with the combined treatment of 5.7 nM paclitaxel with 2.0 mM pirfenidone. The effect of the vehicle at the highest concentration tested in the drug treatments was also evaluated. Results are presented as a % of cell growth and are the mean ± SEM of at least three independent experiments. NS, not significant.

Furthermore, we evaluated whether the combined treatment of paclitaxel with pirfenidone induced a cytotoxic effect against two human non-tumorigenic cell lines, MCF-10A and MCF-12A, using the SRB assay. For that, both cell lines were treated for 48 h with the combined treatment consisting of 5.7 nM paclitaxel and 2.0 mM pirfenidone, as well as with each drug alone. The effect of the vehicle at the highest concentration tested was also evaluated as a negative control. Our results (Figure 7) demonstrated that the drug combination consisting of paclitaxel with pirfenidone did not augment cytotoxicity to both non-tumorigenic cell lines, when compared to paclitaxel treatment alone.

### 2.7. Pirfenidone Sensitizes NCI-H460 Cells to the Combined Treatment of Paclitaxel plus Carboplatin

**Figure 8:**
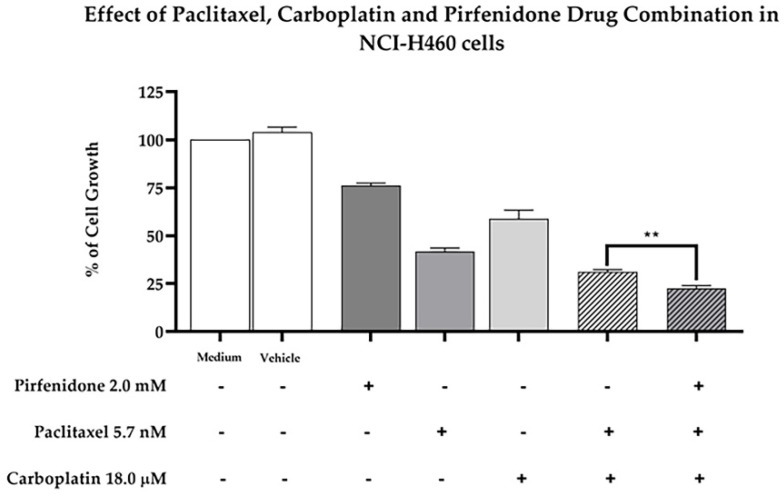
Effect of the combined treatment of paclitaxel and carboplatin with pirfenidone in NCI-H460 cells, assessed by the SRB assay. Cells were treated for 48 h with a drug combination consisting of 5.7 nM paclitaxel, 18 µM carboplatin and 2.0 mM pirfenidone (triplet combination). The effect of the combined treatment consisting of 5.7 nM paclitaxel and 18 µM carboplatin (duplet combination), currently used in clinical practice, was compared with the triplet combination under study. The effect of the vehicle at the highest concentration tested in the drug treatments was also analyzed. Results are presented as a % of cell growth and are the mean ± SEM of at least three independent experiments. ** *p* < 0.01.

Finally, we then analyzed the effect of pirfenidone in sensitizing NCI-H460 cells to the combined treatment of paclitaxel and carboplatin, by measuring the % of cell growth. For that, NCI-H460 cells were treated for 48 h with each drug individually—5.7 nM paclitaxel, 18.0 µM carboplatin and 2.0 mM pirfenidone, with the duplet (paclitaxel and carboplatin), with the triplet (paclitaxel and carboplatin plus pirfenidone) and with the vehicle (at the highest concentration tested in the drug treatments). The obtained results (Figure 8) demonstrated that the triplet drug combination (paclitaxel and carboplatin plus pirfenidone) statistically significantly reduced the % of NCI-H460 cell growth, when compared to treatment with the duplet (paclitaxel and carboplatin). Therefore, our data showed that pirfenidone not only sensitized NCI-H460 cells to paclitaxel treatment, but also to paclitaxel plus carboplatin treatment, both regimens currently used in the clinical practice for NSCLC treatment.

Additionally, we also assessed the ability of pirfenidone to sensitize cells from two NSCLC cell lines (NCI-H460 and A549) to other chemotherapeutic drugs, such as etoposide and gemcitabine, which are also applied in the clinical practice for the treatment of NSCLC (Supplementary Table S1). Our results demonstrated that pirfenidone in combination with etoposide (Supplementary Figure S2) or gemcitabine (Supplementary Figure S3) did not bring advantage over these chemotherapeutic drugs used alone. Moreover, the effect of the combined treatment of pirfenidone with etoposide plus carboplatin (Supplementary Figure S4), which is a duplet commonly applied for the treatment of lung large cell carcinoma, was also evaluated. However, no statistically significant difference was found when comparing this triplet drug combination with the duplet drug combination consisting of etoposide plus carboplatin.

## 3. Discussion

Lung cancer is the world’s deadliest malignancy, and NSCLC is the most frequently diagnosed histologic type [1,2]. Despite recent advances in lung cancer treatment, with the introduction of immune and targeted therapies, the use of these therapeutic modalities as single systemic agent regimens is not suitable for a large number of patients, and chemotherapy remains critical for clinical management [8,10]. Thus, the development of new and more effective therapeutic options such as drug combinations is strictly necessary. Over the past few years, drug repurposing has emerged in oncology as an appealing strategy to identify antitumor potential in drugs already approved or under investigation for the treatment of other diseases [39]. Pirfenidone, a drug approved for the treatment of idiopathic pulmonary fibrosis, has been demonstrated to have antitumor potential as well as to sensitize some tumor models to chemotherapy [22,23,24,25,26,27,28,29,30,31,32,34,35,40]. Importantly, a retrospective study demonstrated that patients with idiopathic pulmonary fibrosis treated with pirfenidone had lower incidences of lung cancer [41]. Therefore, due to the previously described antitumor effect of pirfenidone, our work aimed to evaluate the ability of pirfenidone to sensitize NSCLC cell lines to paclitaxel, a chemotherapeutic drug currently used in clinical practice, with the ultimate aim of contributing to pre-clinical data for the possibility of repurposing pirfenidone in combination with paclitaxel-based regimens for the treatment of NSCLC. 

Our data clearly revealed that the combined treatment of paclitaxel with pirfenidone reduced NCI-H460 cell growth and viability more effectively than treatment with each drug individually, suggesting that pirfenidone sensitizes NCI-H460 cells to paclitaxel treatment. Moreover, this combined drug treatment caused major alterations on the cell cycle profile by reducing the % of NCI-H460 cells in the G2/M phases and increasing the % of cells in the sub-G1 phase. These results suggest that this combined drug treatment caused a decrease in cell proliferation and an increase in cell death. Indeed, these results were further corroborated, since a reduction in cell proliferation, an increase in cell death by apoptosis, and a decrease in the levels of total PARP-1, accompanied by an increase in the levels of cleaved PARP-1 and caspase-3, were observed when NCI-H460 cells were treated with the combination consisting of pirfenidone with paclitaxel.

Our findings are consistent with those reported by Mediavilla-Varela M. et al. (2016), which described a significant reduction in proliferation, accompanied by an increase in cell death, when several NSCLC cell lines were treated with the combined treatment consisting of pirfenidone with cisplatin [35]. Therefore, our data together with the literature suggests that pirfenidone sensitizes NSCLC cell lines to platinum-based chemotherapeutic regimens.

Furthermore, our data corroborates the previously described antitumor potential of pirfenidone treatment alone. Indeed, our work demonstrated that pirfenidone reduces NCI-H460 cell growth, viability and proliferation, and induces a G0/G1 cell cycle arrest. Likewise, in 2019, Ishii K. et al. [23], Usugi E. et al. [24] and Marwitz S. et al. [30] demonstrated the same effects for pirfenidone treatment in prostate, pancreatic and NSCLC cancer models, respectively. In agreement with our results, these authors also found an increase in the % of cells in the G0/G1 phases of the cell cycle accompanied by a reduction in the % of cells in the S phase following pirfenidone treatment alone. 

Importantly, we demonstrated for the first time that the combined treatment of paclitaxel with pirfenidone did not cause additional cytotoxicity against human non-tumorigenic cell lines, when compared with the chemotherapeutic drug used alone. In addition, our work demonstrated that pirfenidone sensitizes NCI-H460 cells to the combined treatment of paclitaxel with carboplatin, which is a chemotherapy duplet currently used in clinical practice for the treatment of NSCLC patients. 

Taken together, our findings provide strong and innovative pre-clinical data to support the possibility of carrying out clinical studies to verify the effect of repurposing pirfenidone in combination with paclitaxel or with paclitaxel plus carboplatin, for the perioperative treatment of NSCLC. To our knowledge, this is the first study exploring the possibility of repurposing pirfenidone in combination with combinatorial platinum-based regimens in vitro. Nevertheless, further pre-clinical studies using complex biological models (e.g., xenografted mice models) must be carried out, to gather enough pre-clinical data to support a clinical trial. 

## 4. Materials and Methods

### 4.1. Drugs

Carboplatin (BP711), doxorubicin (D1515), etoposide (E1383), gemcitabine (G6423), paclitaxel (T7402) and pirfenidone (Y0001769) were purchased from Merck Life Science, Darmstadt, Germany. Paclitaxel, doxorubicin, etoposide and gemcitabine were dissolved in dimethyl-sulfoxide (DMSO; Merck Life Science, Darmstadt, Germany; D2650) at stock concentrations of 1 mM, 2.2 mM, 50 mM and 60 mM, respectively. Carboplatin and pirfenidone were dissolved in sterile water for Molecular Biology (Merck Life Science, Darmstadt, Germany; 95284) at stock concentrations of 26.94 mM and 50 mM, respectively. All drugs were stored at −20 °C, except carboplatin that was stored at 4 °C according to the manufacturer’s instructions.

### 4.2. Cell Culture

Three NSCLC cell lines—A549, NCI-H322 and NCI-H460—and two non-tumorigenic cell lines—MCF-10A and MCF-12A—were used. The NSCLC A549 and NCI-H460 cell lines and the two non-tumorigenic cell lines were purchased from the American Type Culture Collection (ATCC). The NSCLC NCI-H322 cell line was obtained from the European Collection of Authenticated Cell Cultures (ECACC). The A549 cell line was maintained in Dulbecco’s Modified Eagle Medium (DMEM) supplemented with 4.5 g/L Glucose with UltraGlutamine^TM^ w/sodium pyruvate (Lonza, Basel Stücki, Switzerland; BE12-604F), enriched with 10% fetal bovine serum (FBS; Biowest, Nuaillé, France; S181H-500). The NCI-H322 and NCI-H460 cell lines were cultured in Roswell Park Memorial Institute (RPMI) 1640 medium supplemented with Stable Glutamine and 25 mM HEPES (Biowest, Nuaillé, France; L0496-500), complemented with 10% FBS. The non-tumorigenic MCF-10A and MCF-12A cell lines were cultured in DMEM/F12 (Thermo Fischer Scientific, Waltham, MA, USA; 11320033), supplemented with 5% inactivated Horse Serum (HS; Biowest, Nuaillé, France; S0910), 0.5 mg/mL of hydrocortisone (Merck Life Science, Darmstadt, Germany; H0888), 20 ng/mL of human epidermal growth factor (R&D systems, Minneapolis, MI, USA; 236EG), 10 mg/mL of insulin (Merck Life Science, Darmstadt, Germany; I9278), 100 ng/mL of cholera toxin (Merck Life Science, Darmstadt, Germany; C8052), 100 units/mL penicillin and 100 mg/mL of streptomycin solution (100×, Corning Inc., Corning, NY, USA; 30-002-CI), as previously described [42]. For the sulforhodamine B assay, cells were grown in medium supplemented with 5% FBS. Cells were cultured in tissue culture flasks and kept at 37 °C in a humidified chamber containing 5% CO_2_. Routinely, cells were observed using an inverted light microscope (Leica DMi1, Leica Biosystems, Wetzlar, Germany) and they were genotyped and tested for mycoplasma infection. All experiments were carried out with cells at the exponential growth phase and with more than 90% of viability.

### 4.3. Drug Treatments

To determine the GI_50_ concentration of each drug, the NSCLC cell lines were treated for 48 h with: (a) five serial dilutions of the tested drug; (b) vehicle (at the higher concentration used); and (c) medium alone. Carboplatin was tested at concentrations ranging from 6.3 to 100.0 µM; etoposide was tested at concentrations ranging from 0.2 to 10.0 µM; gemcitabine was tested at concentrations ranging from 1.9 × 10^−3^ to 0.1 µM; paclitaxel was tested at concentrations ranging from 4.0 × 10^−4^ to 7.0×10^−2^ µM; and pirfenidone was tested at concentrations ranging from 313.0 to 5.0 × 10^3^ µM.

To evaluate the cytotoxic effect of the combined drug treatments, the NSCLC cell line NCI-H460 was treated for 48 h with two distinct experimental designs: (a) combined treatment of five serial dilutions of etoposide, gemcitabine or paclitaxel with a selected concentration of pirfenidone; or (b) combined treatment of five serial dilutions of pirfenidone with a selected concentration of etoposide, gemcitabine or paclitaxel. The A549 cell line was treated with drug combinations consisting of etoposide, gemcitabine or paclitaxel and a selected concentration of pirfenidone. The concentrations of paclitaxel and pirfenidone that produced the best results for the NCI-H460 cell line were tested in the NCI-H322 cell line. The cytotoxic effect of the combined treatment of 5.7 nM paclitaxel with 2.0 mM pirfenidone against human non-tumorigenic cell lines, MCF-10A and MCF-12A, was evaluated. In all experimental designs, medium alone (blank) and vehicle of the drugs at the highest concentration tested were used as negative controls.

For the following assays, cells were treated for 48 h with: (a) the concentration of each drug alone; (b) the combined drug treatment; (c) vehicle (at the higher concentration tested in drug treatments); and (d) medium alone (blank).

### 4.4. Cell Viability—Trypan Blue Exclusion Assay

Cell number and viability were evaluated using the trypan blue exclusion assay. After drug treatments, cell suspensions were mixed with 0.2% (*v*/*v*) trypan blue dye (Merck Life Science, Darmstadt, Germany; T8154), which distinguishes viable (bright) from non-viable cells (blue ones), at a ratio of 1:1, and the cell number was counted using a hemocytometer (*Neubauer Chamber*).

### 4.5. Cell Growth Inhibition—Sulforhodamine B (SRB) Assay

To determine the concentration of each drug that caused 50% of cell growth inhibition (GI_50_) and to assess the cytotoxic effect of the combined drug treatments, the sulforhodamine B (SRB) assay was performed, according to the described protocol [43]. For that, NSCLC cells were plated in 96-well plates at a previously determined optimal cell concentration (5 × 10^4^ cells/mL) and incubated for 24 h. Then, cells were exposed to the desired drug treatments for 48 h. All studies were conducted in two distinct plates: one to be analyzed at the time of drug treatment (T0) and another to be analyzed 48 h later (T48).

Following a 48-h incubation, cells were fixed with 10% (*w*/*v*) ice-cold trichloroacetic acid (TCA; Merck Life Science, Darmstadt, Germany; T0699) for at least 1 h at 4 °C. After washing cells with distilled water and allowing them to air-dry at room temperature, cells were stained with 0.4% (*w*/*v*) SRB (Merck Life Science, Darmstadt, Germany; S9012) in 1% (*v*/*v*) acetic acid (Merck Life Science, Darmstadt, Germany; T0699) for 30 min, washed with 1% (*v*/*v*) acetic acid to remove the unbound dye and allowed to air-dry at room temperature. The bound SRB was then solubilized with 10 mM Tris base solution in water (Merck Life Science, Darmstadt, Germany; T6066) and absorbance was measured at 510 nm in a multi plate reader (Synergy^TM^ Mx, Biotek Instruments Inc., Winooski, VT, USA), recurring to the Gen5^TM^ software.

### 4.6. Cell Proliferation—5-Bromo-2′-Deoxyuridine (BrdU) Incorporation Assay

Cell proliferation was assessed using the 5-bromo-2′-deoxyuridine (BrdU) incorporation assay, according to the described protocol [37,44,45]. For that, NCI-H460 cells were plated in 6-well plates at a previously determined optimal cell concentration (5 × 10^4^ cells/mL) and incubated for 24 h. Then, cells were subjected to the desired treatments for 48 h. Nearly 4 h before cell collection, cells of each condition were incubated with BrdU (Merck Life Science, Darmstadt, Germany; B5002) at a final concentration of 10 μM. After collection, cells were centrifuged (1200 rpm for 5 min at room temperature), washed with phosphate buffered saline (PBS; Merck Life Science, Darmstadt, Germany; P5493) and fixed with paraformaldehyde 4% (PFA; Merck Life Science, Darmstadt, Germany; 1.04005) for 40 min at room temperature. Then, cells were subjected to two sequential centrifugations under the same previously described conditions, resuspended in PBS and stored at 4 °C. Following cytospin preparation, cellular DNA denaturation was induced by treatment with 2 M HCl (Merck Life Science, Darmstadt, Germany; 258148) for 20 min and blockage was performed with a PBS with 0.5% Tween (Promega, Madison, WI, USA; H5152) and 0.05% Bovine Serum Albumin (BSA; Merck Life Science, Darmstadt, Germany; A7906) solution. Then, cells were incubated with the monoclonal mouse anti-BrdU Clone Bu20a primary antibody (1:10; Dako, Glostrup, Denmark; M0744) for 1 h, followed by incubation with the polyclonal rabbit anti-mouse immunoglobulins/FITC secondary antibody (1:100; Dako, Glostrup, Denmark; F0261) for 30 min. After that, slides were prepared with vectashield mounting medium containing 4′,6-diamidino-2-phenylindole (DAPI; Vector Laboratories Inc., Peterborough, UK; H-1200). The detection of BrdU incorporation was possible using the Zeiss Axio Imager Z1 (Carl Zeiss, Jena, Germany) microscope and the Axiovision 4.9 (Carl Zeiss, Jena, Germany) software and the evaluation of the % of proliferating cells was performed by counting a minimum of 200 cells per slide using the ImageJ 2.1.0 software.

### 4.7. Cell Cycle Profile—Flow Cytometry following Propidium Iodide (PI) Staining

Cell cycle profile was assessed by flow cytometry following propidium iodide (PI) staining, according to the described protocol [37,38,44,45]. For that, NCI-H460 cells were plated in 6-well plates at a previously determined optimal cell concentration (5 × 10^4^ cells/mL) and incubated for 24 h. Then, cells were treated with the desired conditions for 48 h. After the incubation period, cells of each condition were collected, centrifuged (1200 rpm for 5 min at 4 °C) and fixed with ice-cold 70% ethanol (Fischer Scientific, Hampton, NH, USA; E/0650DF/C17) at 4 °C for a period of at least 12 h. Then, cells were centrifuged at the previously described conditions and each cell pellet was resuspended in a PBS solution containing 0.1 mg/mL RNase A (Invitrogen, Waltham, MA, USA; 12091021) and 5 µg/mL PI (Merck Life Science, Darmstadt, Germany; 537060) and kept in the dark for at least 30 min. Sample analysis was performed using the BD Accuri™ C6 Flow Cytometer (BD Biosciences, San Jose, CA, USA) and the BDSamples software (BD Biosciences, San Jose, CA, USA), and after the proper exclusion of cell debris and aggregates, at least 10,000 to 20,000 events per sample were plotted. Results were further analyzed using the FlowJo 7.6.5 Software (Tree Star Inc., San Carlos, CA, USA).

### 4.8. Cell Death—Annexin V-FITC Apoptosis Detection Assay

Cell death was assessed using the Annexin V-FITC Apoptosis Detection Kit (eBioscience™, Thermo Fisher Scientific, Waltham, MA, USA; BMS500FI), according to manufacturer’s instructions and to the previously described protocol [37,38,45]. For that, NCI-H460 cells were plated in 6-well plates, and incubated for 24 h. Then, cells were exposed to the desired drug treatments for 48 h. Nearly 1 h before cell collection, pure ethanol was added to the well corresponding to the positive control for necrosis. After collection, cells were centrifuged for 5 min at 1200 rpm at 4 °C and resuspended in the binding buffer solution in water (1×). As indicated by the manufacturer’s instructions, cell suspensions were then incubated with the Annexin V-FITC conjugate for 10 min at room temperature, protected from light. Subsequently, PI was added and samples were analyzed using the BD Accuri™ C6 Flow Cytometer and the BDSamples software; after the proper exclusion of cell debris and aggregates, at least 10,000 to 20,000 events per sample were plotted. The flow cytometry results were further analyzed using the BD Accuri^TM^ C6 Software (BD Biosciences, San Jose, CA, USA).

### 4.9. Protein Expression Analysis—Protein Extraction and Western Blotting

For protein expression analysis, NCI-H460 cells were plated in 6-well plates, and incubated for 24 h. Then, cells were treated with the selected drugs. After a 48 h incubation period, cell pellets were washed with PBS by centrifugation at 1000 rpm for 5 min at 4 °C, and lysed in Wynman’s buffer (1% NP-40 (Merck Life Science, Darmstadt, Germany; 74385), 0.1 M Tris-HCl pH 8.0 (Merck Life Science, Darmstadt, Germany; T-7149), 0.15 M NaCl (Merck Life Science, Darmstadt, Germany; S3014), 5 mM EDTA (Merck Life Science, Darmstadt, Germany; E0399) complemented with protease inhibitor cocktail (Roche, 11836145001) and phosphatase inhibitor (Merck Life Science, Darmstadt, Germany; S6508), for 30 min at 4 °C. Protein lysates were obtained after centrifugation at 13,000 rpm for 10 min at 4 °C and quantified using a modified Lowry protocol (DC™ Protein Assay kit; Bio-Rad, Hercules, CA, USA; 5000116). BSA was used as a protein standard. A total of 25 µg of protein lysates corresponding to each experimental condition were loaded and separated in a 10% SDS-Page gel for 30 min at 70 V and for approximately 1 h at 100 V, and transferred to a nitrocellulose membrane (GE Healthcare Life science, Chalfont St Giles, UK; GE10600002) for 1 h at 100 V. Membranes were stained with Ponceau S solution (PanReac AppliChem, Barcelona, Spain; A2935) to confirm protein transfer and blocked in Tris-buffered saline solution (TBS) pH 7.4 (80 g NaCl, 2 g KCl (Merck Life Science, Darmstadt, Germany; P9541), 30 g Tris-Base) solution with 0.1% Tween^®^ 20 (Promega, H5152)—TBS-T—containing either 5% (*w*/*v*) non-fat dry milk (Molico, Nestlé, Vevey, Switzerland) or 5% (*w*/*v*) BSA (in the case of phosphorylated proteins) for 30 minutes at room temperature, in an orbital shaker. Membranes were then incubated overnight with agitation at 4 °C with the following primary antibodies: mouse anti-PARP-1 (F-2) (sc-8007) and mouse anti-caspase-3 (31A1067) (1:200; sc-56053), from Santa Cruz Biotechnology, Dallas, TX, USA. After washing steps with TBS-T, membranes were incubated with the secondary antibody goat anti-mouse IgG-HRP (1:2000; sc-2031), from Santa Cruz Biotechnology, for 1 h at room temperature, in an orbital shaker. After washing steps with TBS-T, peroxidase activity was revealed in Amersham Hyperfilm ECL (Cytiva, Marlborough, MA, USA; 28906835), using the ECL™ Western Blotting detection reagents (Cytiva, Marlborough, MA, USA; RPN2209) and signal from the membranes was detected using Fujifilm™ Anatomix X-Ray System developer (Fujifilm, Tokyo, Japan; XC914093) and fixer (Fujifilm, Tokyo, Japan; XC914135). Immunoblots were digitalized using the GS-800 Calibrated Densitometer (Bio-Rad, Hercules, CA, USA; 170-7980).

### 4.10. Statistical Analysis

All experiments were performed at least three independent times, and results are expressed as mean ± SEM. The statistical analysis was performed using the two-tailed unpaired *t*-test, with GraphPad Prism 8.0 software. Statistical significance was considered whenever *p* < 0.05.

## Figures and Tables

**Table 1:** GI_50_ concentrations (µM) of paclitaxel and pirfenidone in three non-small cell lung cancer (NSCLC) cell lines.

	GI_50_ * Concentration (µM) for each NSCLC Cell Line		
Drug	A549	NCI-H322	NCI-H460
Paclitaxel	2.7 × 10^−3^ ± 0.0	4.7 × 10^−3^ ± 4.7 × 10^−4^	5.7 × 10^−3^ ± 5.3 × 10^−4^
Pirfenidone	1.5 × 10^3^ ± 1.9 × 10^2^	2.8 × 10^3^ ± 1.5 × 10^2^	1.9 × 10^3^ ± 3.0 × 10^1^

* Concentration that causes 50% cell growth inhibition (GI_50_) determined 48 h following drug treatment, using the SRB assay. Results are the mean ± SEM of at least three independent experiments.

## Supplementary Materials

The following supporting information can be downloaded at: https://www.mdpi.com/article/10.3390/ijms23073631/s1.

## References

[1] Sung H., Ferlay J., Siegel R.L., Laversanne M., Soerjomataram I., Jemal A., Bray F. (2021). Global Cancer Statistics 2020: GLOBOCAN Estimates of Incidence and Mortality Worldwide for 36 Cancers in 185 Countries. CA Cancer J. Clin..

[2] Duma N., Santana-Davila R., Molina J.R. (2019). Non-Small Cell Lung Cancer: Epidemiology, Screening, Diagnosis, and Treatment. Mayo Clin. Proc..

[3] Lemjabbar-Alaoui H., Hassan O.U., Yang Y.W., Buchanan P. (2015). Lung cancer: Biology and treatment options. Biochim. Biophys. Acta.

[4] Gridelli C., Rossi A., Carbone D.P., Guarize J., Karachaliou N., Mok T., Petrella F., Spaggiari L., Rosell R. (2015). Non-small-cell lung cancer. Nat. Rev. Dis. Primers.

[5] Burdett S., Pignon J.P., Tierney J., Tribodet H., Stewart L., Le Pechoux C., Aupérin A., Le Chevalier T., Stephens R.J., Arriagada R. (2015). Adjuvant chemotherapy for resected early-stage non-small cell lung cancer. Cochrane Database Syst. Rev..

[6] (2014). Preoperative chemotherapy for non-small-cell lung cancer: A systematic review and meta-analysis of individual participant data. Lancet.

[7] Nooreldeen R., Bach H. (2021). Current and Future Development in Lung Cancer Diagnosis. Int. J. Mol. Sci..

[8] Wang M., Herbst R.S., Boshoff C. (2021). Toward personalized treatment approaches for non-small-cell lung cancer. Nat. Med..

[9] Reck M., Rodríguez-Abreu D., Robinson A.G., Hui R., Csőszi T., Fülöp A., Gottfried M., Peled N., Tafreshi A., Cuffe S. (2021). Five-Year Outcomes With Pembrolizumab Versus Chemotherapy for Metastatic Non-Small-Cell Lung Cancer with PD-L1 Tumor Proportion Score ≥ 50. J. Clin. Oncol. Off. J. Am. Soc. Clin. Oncol..

[10] Planchard D., Popat S., Kerr K., Novello S., Smit E.F., Faivre-Finn C., Mok T.S., Reck M., Van Schil P.E., Hellmann M.D. (2018). Metastatic non-small cell lung cancer: ESMO Clinical Practice Guidelines for diagnosis, treatment and follow-up. Ann. Oncol. Off. J. Eur. Soc. Med. Oncol..

[11] Rotow J., Bivona T.G. (2017). Understanding and targeting resistance mechanisms in NSCLC. Nat. Rev. Cancer.

[12] Błach J., Wojas-Krawczyk K., Nicoś M., Krawczyk P. (2021). Failure of Immunotherapy-The Molecular and Immunological Origin of Immunotherapy Resistance in Lung Cancer. Int. J. Mol. Sci..

[13] Reita D., Pabst L., Pencreach E., Guérin E., Dano L., Rimelen V., Voegeli A.C., Vallat L., Mascaux C., Beau-Faller M. (2021). Molecular Mechanism of EGFR-TKI Resistance in EGFR-Mutated Non-Small Cell Lung Cancer: Application to Biological Diagnostic and Monitoring. Cancers.

[14] Ashburn T.T., Thor K.B. (2004). Drug repositioning: Identifying and developing new uses for existing drugs. Nat. Rev. Drug Discov..

[15] Langedijk J., Mantel-Teeuwisse A.K., Slijkerman D.S., Schutjens M.H. (2015). Drug repositioning and repurposing: Terminology and definitions in literature. Drug Discov. Today.

[16] Pushpakom S., Iorio F., Eyers P.A., Escott K.J., Hopper S., Wells A., Doig A., Guilliams T., Latimer J., McNamee C. (2019). Drug repurposing: Progress, challenges and recommendations. Nat. Rev. Drug Discov..

[17] Gupta S.C., Sung B., Prasad S., Webb L.J., Aggarwal B.B. (2013). Cancer drug discovery by repurposing: Teaching new tricks to old dogs. Trends Pharmacol. Sci..

[18] Rebelo R., Polónia B., Santos L.L., Vasconcelos M.H., Xavier C.P.R. (2021). Drug Repurposing Opportunities in Pancreatic Ductal Adenocarcinoma. Pharmaceuticals.

[19] Cha Y., Erez T., Reynolds I.J., Kumar D., Ross J., Koytiger G., Kusko R., Zeskind B., Risso S., Kagan E. (2018). Drug repurposing from the perspective of pharmaceutical companies. Br. J. Pharmacol..

[20] Nosengo N. (2016). Can you teach old drugs new tricks?. Nature.

[21] Taniguchi H., Ebina M., Kondoh Y., Ogura T., Azuma A., Suga M., Taguchi Y., Takahashi H., Nakata K., Sato A. (2010). Pirfenidone in idiopathic pulmonary fibrosis. Eur. Respir. J..

[22] King T.E., Bradford W.Z., Castro-Bernardini S., Fagan E.A., Glaspole I., Glassberg M.K., Gorina E., Hopkins P.M., Kardatzke D., Lancaster L. (2014). A phase 3 trial of pirfenidone in patients with idiopathic pulmonary fibrosis. N. Engl. J. Med..

[23] Ishii K., Sasaki T., Iguchi K., Kato M., Kanda H., Hirokawa Y., Arima K., Watanabe M., Sugimura Y. (2019). Pirfenidone, an Anti-Fibrotic Drug, Suppresses the Growth of Human Prostate Cancer Cells by Inducing G₁ Cell Cycle Arrest. J. Clin. Med..

[24] Usugi E., Ishii K., Hirokawa Y., Kanayama K., Matsuda C., Uchida K., Shiraishi T., Watanabe M. (2019). Antifibrotic Agent Pirfenidone Suppresses Proliferation of Human Pancreatic Cancer Cells by Inducing G0/G1 Cell Cycle Arrest. Pharmacology.

[25] Zou W.J., Huang Z., Jiang T.P., Shen Y.P., Zhao A.S., Zhou S., Zhang S. (2017). Pirfenidone Inhibits Proliferation and Promotes Apoptosis of Hepatocellular Carcinoma Cells by Inhibiting the Wnt/β-Catenin Signaling Pathway. Med. Sci. Monit. Int. Med. J. Exp. Clin. Res..

[26] Li C., Rezov V., Joensuu E., Vartiainen V., Rönty M., Yin M., Myllärniemi M., Koli K. (2018). Pirfenidone decreases mesothelioma cell proliferation and migration via inhibition of ERK and AKT and regulates mesothelioma tumor microenvironment in vivo. Sci. Rep..

[27] Burghardt I., Tritschler F., Opitz C.A., Frank B., Weller M., Wick W. (2007). Pirfenidone inhibits TGF-beta expression in malignant glioma cells. Biochem. Biophys. Res. Commun..

[28] Kozono S., Ohuchida K., Eguchi D., Ikenaga N., Fujiwara K., Cui L., Mizumoto K., Tanaka M. (2013). Pirfenidone inhibits pancreatic cancer desmoplasia by regulating stellate cells. Cancer Res..

[29] Aboulkheyr Es H., Zhand S., Thiery J.P., Warkiani M.E. (2020). Pirfenidone reduces immune-suppressive capacity of cancer-associated fibroblasts through targeting CCL17 and TNF-beta. Integr. Biol. Quant. Biosci. Nano Macro.

[30] Marwitz S., Turkowski K., Nitschkowski D., Weigert A., Brandenburg J., Reiling N., Thomas M., Reck M., Drömann D., Seeger W. (2019). The Multi-Modal Effect of the Anti-fibrotic Drug Pirfenidone on NSCLC. Front. Oncol..

[31] Fujiwara A., Shintani Y., Funaki S., Kawamura T., Kimura T., Minami M., Okumura M. (2017). Pirfenidone plays a biphasic role in inhibition of epithelial-mesenchymal transition in non-small cell lung cancer. Lung Cancer.

[32] Kurimoto R., Ebata T., Iwasawa S., Ishiwata T., Tada Y., Tatsumi K., Takiguchi Y. (2017). Pirfenidone may revert the epithelial-to-mesenchymal transition in human lung adenocarcinoma. Oncol. Lett..

[33] Choi S.H., Nam J.K., Jang J., Lee H.J., Lee Y.J. (2015). Pirfenidone enhances the efficacy of combined radiation and sunitinib therapy. Biochem. Biophys. Res. Commun..

[34] Polydorou C., Mpekris F., Papageorgis P., Voutouri C., Stylianopoulos T. (2017). Pirfenidone normalizes the tumor microenvironment to improve chemotherapy. Oncotarget.

[35] Mediavilla-Varela M., Boateng K., Noyes D., Antonia S.J. (2016). The anti-fibrotic agent pirfenidone synergizes with cisplatin in killing tumor cells and cancer-associated fibroblasts. BMC Cancer.

[36] Qin W., Zou J., Huang Y., Liu C., Kang Y., Han H., Tang Y., Li L., Liu B., Zhao W. (2020). Pirfenidone facilitates immune infiltration and enhances the antitumor efficacy of PD-L1 blockade in mice. Oncoimmunology.

[37] Teixeira A., DaCunha D.C., Barros L., Caires H.R., Xavier C.P.R., Ferreira I., Vasconcelos M.H. (2019). Eucalyptus globulus Labill. decoction extract inhibits the growth of NCI-H460 cells by increasing the p53 levels and altering the cell cycle profile. Food Funct..

[38] Magalhães D.B., Castro I., Lopes-Rodrigues V., Pereira J.M., Barros L., Ferreira I., Xavier C.P.R., Vasconcelos M.H. (2018). Melissa officinalis L. ethanolic extract inhibits the growth of a lung cancer cell line by interfering with the cell cycle and inducing apoptosis. Food Funct..

[39] Schein C.H. (2021). Repurposing approved drugs for cancer therapy. Br. Med. Bull..

[40] Krämer M., Markart P., Drakopanagiotakis F., Mamazhakypov A., Schaefer L., Didiasova M., Wygrecka M. (2020). Pirfenidone inhibits motility of NSCLC cells by interfering with the urokinase system. Cell. Signal..

[41] Miura Y., Saito T., Tanaka T., Takoi H., Yatagai Y., Inomata M., Nei T., Saito Y., Gemma A., Azuma A. (2018). Reduced incidence of lung cancer in patients with idiopathic pulmonary fibrosis treated with pirfenidone. Respir. Investig..

[42] Long S., Resende D., Kijjoa A., Silva A.M.S., Fernandes R., Xavier C.P.R., Vasconcelos M.H., Sousa E., Pinto M.M.M. (2019). Synthesis of New Proteomimetic Quinazolinone Alkaloids and Evaluation of Their Neuroprotective and Antitumor Effects. Molecules.

[43] Vichai V., Kirtikara K. (2006). Sulforhodamine B colorimetric assay for cytotoxicity screening. Nat. Protoc..

[44] Silva B.R., Rebelo R., Rodrigues J.M., Xavier C.P.R., Vasconcelos M.H., Queiroz M.R.P. (2021). Synthesis of Novel Methyl 3-(hetero)arylthieno[3,2-b]pyridine-2-carboxylates and Antitumor Activity Evaluation: Studies In Vitro and In Ovo Grafts of Chick Chorioallantoic Membrane (CAM) with a Triple Negative Breast Cancer Cell Line. Molecules.

[45] Bizarro A., Sousa D., Lima R.T., Musso L., Cincinelli R., Zuco V., De Cesare M., Dallavalle S., Vasconcelos M.H. (2018). Synthesis and Evaluation of the Tumor Cell Growth Inhibitory Potential of New Putative HSP90 Inhibitors. Molecules.

