# Supplementary material for: Pirfenidone Sensitizes NCI-H460 Non-Small Cell Lung Cancer Cells to Paclitaxel and to a Combination of Paclitaxel with Carboplatin
Source: Int J Mol Sci. 2022 Mar 26;23(7):3631. doi: 10.3390/ijms23073631 (PMC8998757; doi:10.3390/ijms23073631)
Supplement: Supplementary file 1 [file ijms-23-03631-s001.zip › ijms-1644374 supplementary.pdf]

International Journal of
Molecular Sciences

MDPI

Supplementary Data

Pirfenidone sensitizes NCI-H460 non-small cell lung cancer cells to paclitaxel and to a combination of paclitaxel with carboplatin

Helena Branco [1,2], Júlio Oliveira [3], Catarina Antunes [1,2], Lúcio L. Santos [3,4], M. Helena Vasconcelos [1,2,5,*], Cristina P.R. Xavier [1,2,*]

1 i3S – Instituto de Investigação e Inovação em Saúde, Universidade do Porto, Rua Alfredo Allen 208, 4200-135 Porto, Portugal. hbranco@ipatimup.pt (H.B.); catarina.teixeira.antunes@gmail.com (C.A.)

2 Cancer Drug Resistance Group, IPATIMUP – Institute of Molecular Pathology and Immunology of the University of Porto, Rua Alfredo Allen 208, 4200-135 Porto, Portugal.

3 Experimental Pathology and Therapeutics Group, IPO – Instituto Português de Oncologia, Rua Dr. António Bernardino de Almeida 865, 4200-072 Porto, Portugal. julio.oliveira@ipoporto.min-saude.pt (J.O.); lucios@ufp.pt (L.L.S.)

4 ICBAS-UP – School of Medicine and Biomedical Sciences, University of Porto, Rua de Jorge Viterbo Ferreira 228, 4050-313 Porto, Portugal.

5 Department of Biological Sciences, FFUP – Faculty of Pharmacy, University of Porto, Rua de Jorge Viterbo Ferreira 228, 4050-313 Porto, Portugal.

* Correspondence: hvasconcelos@ipatimup.pt (M.H.V.) and cristinax@ipatimup.pt (C.P.R.X.); Tel. +351 225 570 772 (M.H.V.).

Supplementary Figures

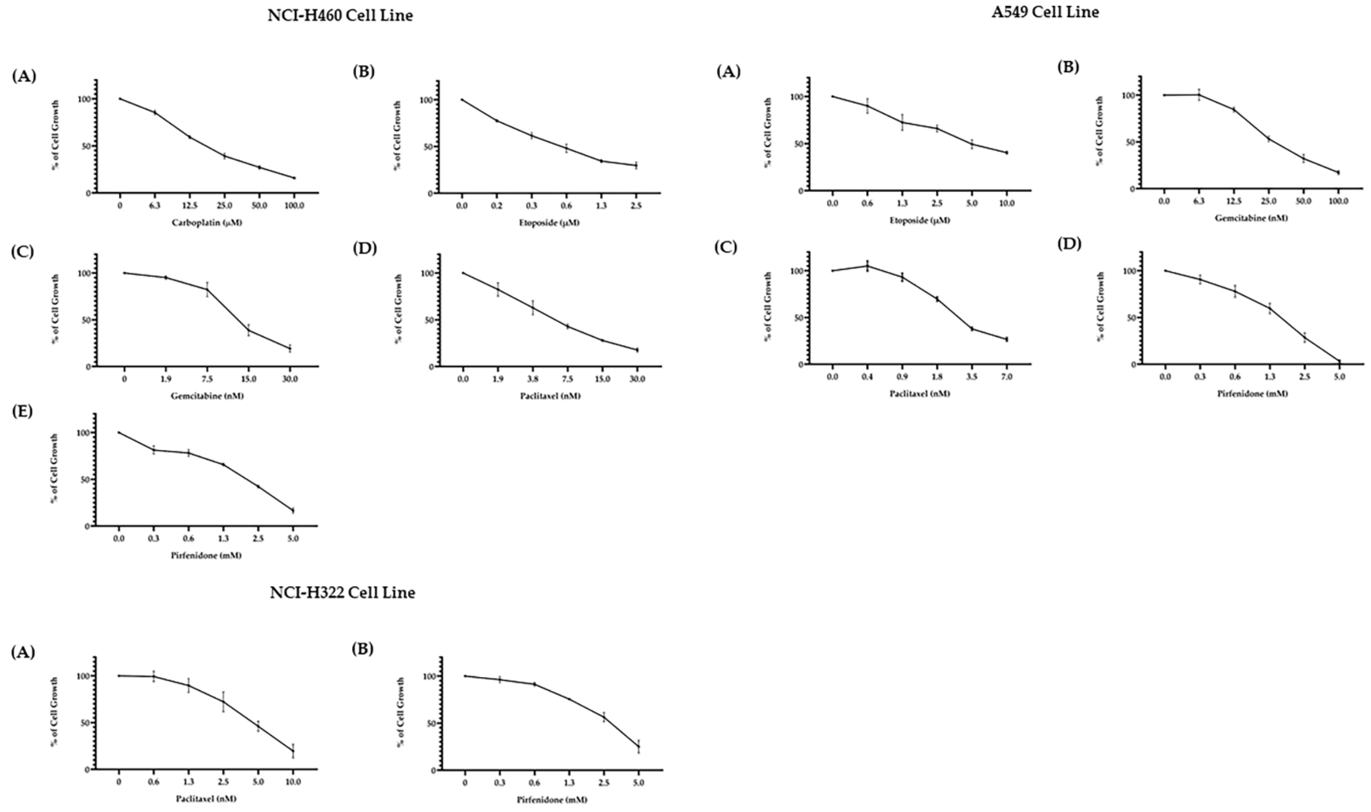

Supplementary Figure S1. Dose-response curves of the NCI-H460, A549 and NCI-H322 cell lines. Cells were treated with five serial dilutions of each individual drug for 48 h and results were obtained with the SRB assay. Results are presented as a % of cell growth and are the mean ± SEM of at least three independent experiments.

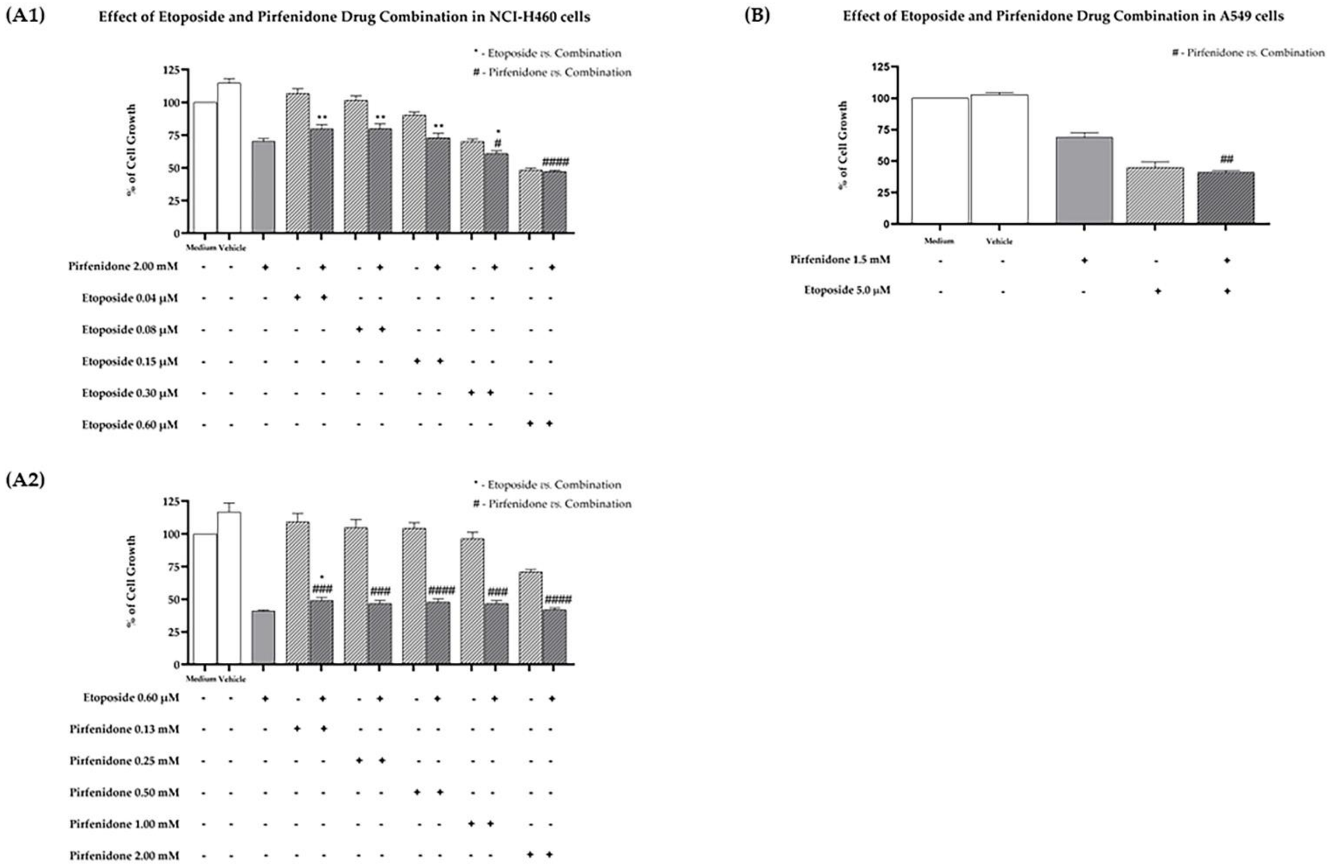

Supplementary Figure S2. Effect of the combined treatment with etoposide and pirfenidone on the % of cell growth of NCI-H460 and A549 cells, assessed by the SRB assay. NCI-H460 cells treated for 48 h with drug combinations consisting of (*A1*) 2.0 mM pirfenidone and five serial dilutions of etoposide; or (**A2**) 0.6 µM etoposide with five serial dilutions of pirfenidone. A549 cells treated for 48 h with a drug combination consisting of 1.5 mM pirfenidone and 5.0 µM etoposide **(B)**. The effect of the vehicle at the highest concentration tested was also analyzed. Results are presented as a % of cell growth and are the mean ± SEM of at least three independent experiments. * or # $p < 0.05$, ## $p < 0.01$, ### $p < 0.001$ and #### $p < 0.0001$.

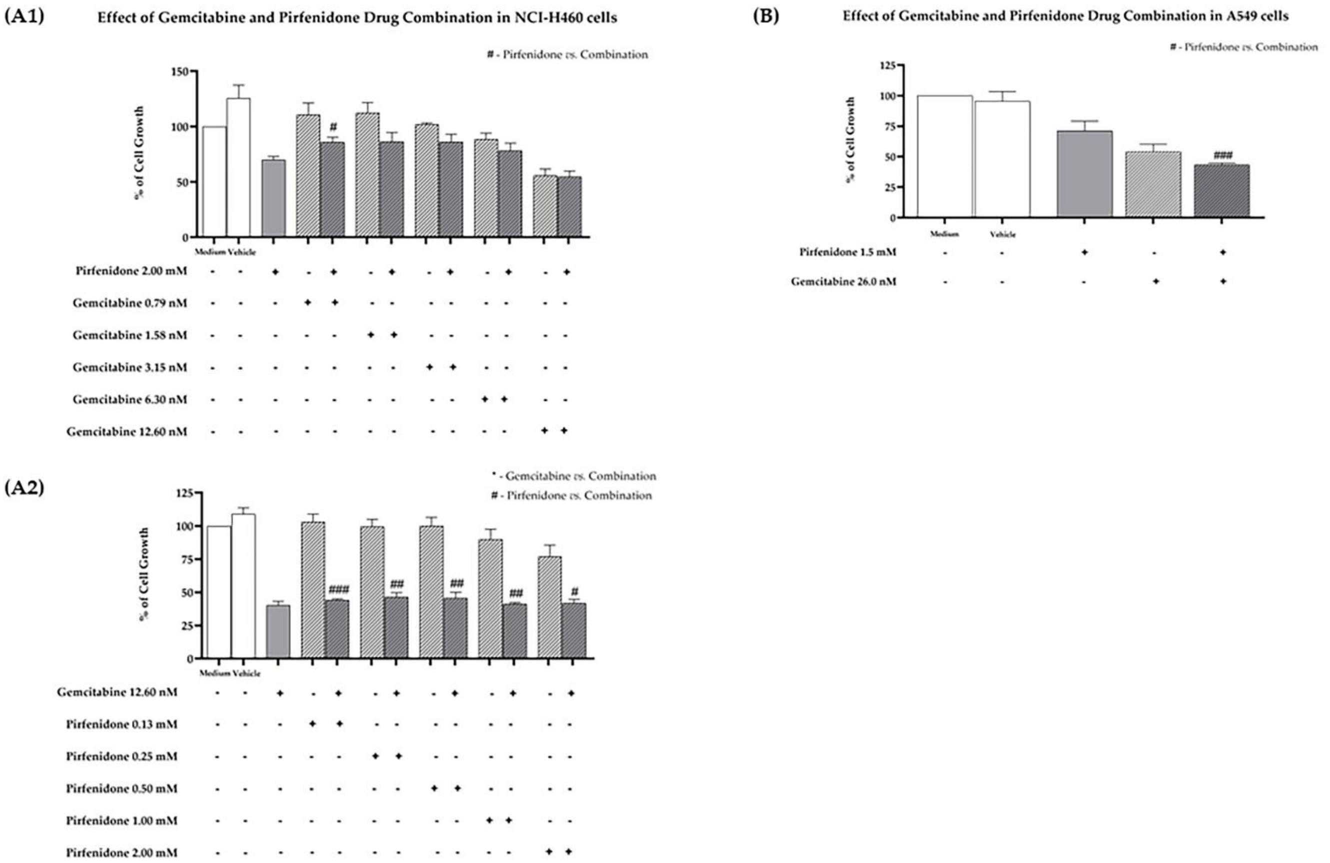

Supplementary Figure S3. Effect of the combined treatment with gemcitabine and pirfenidone on the % of cell growth of NCI-H460 and A549 cells, assessed by the SRB assay. NCI-H460 cells treated for 48 h with drug combinations consisting of **(A1)** 2.0 mM pirfenidone with five serial dilutions of gemcitabine; or **(A2)** 12.6 nM gemcitabine with five serial dilutions of pirfenidone. A549 cells treated for 48 h with a drug combination consisting of 1.5 mM pirfenidone and 12.6 nM gemcitabine **(B)**. The effect of the vehicle at the highest concentration tested in the drug treatments was also analyzed. Results are presented as a % of cell growth and are the mean ± SEM of at least three independent experiments. # $p < 0.05$, ## $p < 0.01$ and ### $p < 0.001$.

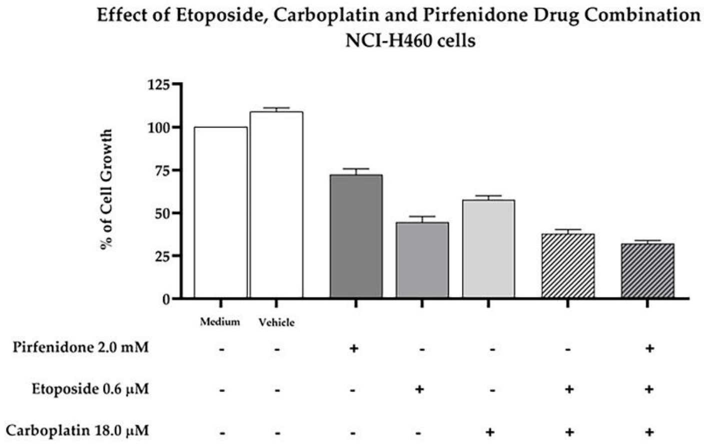

Supplementary Figure S4. Effect of the combined treatment with etoposide and carboplatin plus pirfenidone in NCI-H460 cells, assessed by the SRB assay. Cells were treated for 48 h with a drug combination consisting of 0.6 μM etoposide, 18 μM carboplatin and 2.0 mM pirfenidone. The effect of the combined treatment consisting of 0.6 μM etoposide and 18 μM carboplatin, used in the clinical practice, was compared with the triplet under study. The effect of the vehicle at the highest concentration tested was also analyzed. Results are presented as a % of cell growth and are the mean ± SEM of at least three independent experiments.

Supplementary Table

Supplementary Table S1. GI_{50} concentration (μM) of carboplatin, etoposide and gemcitabine in two NSCLC cancer cell lines.

Drug	A549	NCI-H460
Carboplatin	-	17.7 ± 1.2
Etoposide	5.2 ± 0.8	0.6 ± 0.1
Gemcitabine	$26.3 \times 10^{-3} \pm 1.8 \times 10^{-3}$	$12.6 \times 10^{-3} \pm 1.3 \times 10^{-3}$

* The concentration that causes 50% cell growth inhibition (GI_{50}) of etoposide and gemcitabine in A549 and NCI-H460 human NSCLC cell lines, determined 48 h following drug treatment, using the SRB assay. Results are the mean ± SEM of at least three independent experiments.
